# A Closer Look at the Effects of Repeated Cocaine Exposure on Adaptive Decision-Making under Conditions That Promote Goal-Directed Control

**DOI:** 10.3389/fpsyt.2016.00044

**Published:** 2016-03-21

**Authors:** Briac Halbout, Angela T. Liu, Sean B. Ostlund

**Affiliations:** ^1^Department of Anesthesiology and Perioperative Care, School of Medicine, University of California Irvine, Irvine, CA, USA; ^2^UC Irvine Center for Addiction Neuroscience, Irvine, CA, USA

**Keywords:** habit learning, contingency degradation, outcome devaluation, rat, goal-directed, sensitization, choice, cognitive control

## Abstract

It has been proposed that compulsive drug seeking reflects an underlying dysregulation in adaptive behavior that favors habitual (automatic and inflexible) over goal-directed (deliberative and highly flexible) action selection. Rodent studies have established that repeated exposure to cocaine or amphetamine facilitates the development of habits, producing behavior that becomes unusually insensitive to a reduction in the value of its outcome. The current study more directly investigated the effects of cocaine pre-exposure on goal-directed learning and action selection using an approach that discourages habitual performance. After undergoing a 15-day series of cocaine (15 or 30 mg/kg, i.p.) or saline injections and a drug withdrawal period, rats were trained to perform two different lever-press actions for distinct reward options. During a subsequent outcome devaluation test, both cocaine- and saline-treated rats showed a robust bias in their choice between the two actions, preferring whichever action had been trained with the reward that retained its value. Thus, it appears that the tendency for repeated cocaine exposure to promote habit formation does not extend to a more complex behavioral scenario that encourages goal-directed control. To further explore this issue, we assessed how prior cocaine treatment would affect the rats’ ability to learn about a selective reduction in the predictive relationship between one of the two actions and its outcome, which is another fundamental feature of goal-directed behavior. Interestingly, we found that cocaine-treated rats showed enhanced, rather than diminished, sensitivity to this action–outcome contingency degradation manipulation. Given their mutual dependence on striatal dopamine signaling, we suggest that cocaine’s effects on habit formation and contingency learning may stem from a common adaptation in this neurochemical system.

## Introduction

For many, recreational drug use can develop into a pathological behavior that is difficult to control or abstain from despite its many harmful consequences. Similarly, when rodents are given extensive opportunity to self-administer cocaine, they can develop a compulsive tendency to seek out the drug even when doing so leads to physical punishment (1, 2). Understanding how this pathological decision-making develops is a major objective of addiction research and theory.

Some have proposed that compulsive tendencies are caused by drug-induced dysregulation of neural systems that normally mediate adaptive reward-related learning and decision-making (3–8). Although this hypothesis draws heavily on literature regarding animal learning, current evidence shows that humans and rodents use analogous action selection strategies when pursuing rewards (9–13). For instance, when first encountering a task or problem, both species tend to apply a sophisticated goal-directed strategy that allows for rapid learning and flexible decision-making. The term *goal-directed*, here, refers to a reward-seeking action that is performed because an individual infers that doing so will lead to a desired outcome, as opposed to automatically performing an action that has become habitual or routine. One way to determine if an action is goal-directed is to change the value of its outcome between initial training and testing. For instance, rats trained to perform a lever-press action for food pellets will withhold this behavior if they are fed to satiety on those food pellets (instead of some other type of food) immediately before the test session (9, 14, 15). Importantly, outcome devaluation tests are conducted in extinction to ensure that changes in performance are based on previously encoded action–outcome learning.

Another test of goal-directed performance involves changing the causal relationship between an action and its outcome. The contingency degradation procedure accomplishes this by delivering the outcome with the same probability regardless of whether an action is performed or not. In such studies, rats trained to lever press for food pellets will exhibit a decline in this behavior if it is no longer needed to produce pellets (9, 16, 17).

Because goal-directed control involves executive processes that tax cognitive resources (18), both rodents and humans tend to shift to a more efficient, but less flexible, habit-based strategy when appropriate. For instance, rats given extensive training on a simple task tend to be insensitive to manipulations of outcome value or action–outcome contingency (10, 14). Relying on a habitual action selection strategy allows an individual to automatically perform routine reward-seeking tasks while freeing up cognitive resources for other activities.

Based on this conceptual framework, it has been suggested that neuroadaptations caused by chronic drug intake bias action selection in favor of habitual control of drug and adaptive reward seeking (3–5, 7, 19). In line with this general account, there have been many reports that drug and alcohol seeking become insensitive to post-training outcome devaluation (or related treatments), particularly after extensive training (20–24). Those studies, aimed at modeling a loss of control over volitional, drug-directed, actions have shown that initial drug taking can become habitual with prolonged drug use. Interestingly, there is further evidence that the impact of chronic drug experience (volitional or not) on behavioral control is so profound that it even alters the way animals pursue other non-drug rewards. For example, rats given repeated exposure to cocaine or amphetamine before learning to lever press for food reward develop habitual (devaluation insensitive) performance under limited-training conditions that support goal-directed performance in drug-naive rats (25–29).

It is important to note, however, that under normal conditions the transition from goal-directed to habitual performance is neither final nor mandatory. For instance, normal individuals tend to rapidly re-exert goal-directed control over habitual actions if they encounter response-contingent punishment or other salient stimuli (4, 18). Of even greater relevance to the current study, it is known that certain training factors discourage the transition to habitual control. For instance, rats trained with multiple action–outcome relationships typically maintain goal-directed performance even after extensive training (30–34), presumably because executive processes continue to be engaged in settings that encourage consideration of distinct action–outcome relationships (13, 35, 36).

With this in mind, it is interesting that most studies investigating if drug pre-exposure disrupts the balance between goal-directed and habitual control have used simple reward-seeking tasks that would normally support habitual performance in drug-naive animals if sufficient training were provided. Although such findings indicate that chronic drug exposure can facilitate the development of habits, they do not address whether it also compromises goal-directed control in more complex decision-making scenarios that require choice between different response options. This is significant because, for human addicts, the decision to use drugs would seem to occur in situations where countless other more adaptive activities are available. Interestingly, of the few animal studies that have addressed this issue, there is evidence that certain aspects of goal-directed behavior may be unimpaired (28, 37, 38), or perhaps even enhanced (39, 40), following repeated drug exposure.

The current study tests this hypothesis by giving rats repeated experimenter-administered injections of saline or cocaine prior to training them on a challenging instrumental learning protocol involving two distinct action–outcome contingencies. Their ability to exert goal-directed control over task performance was then assessed using outcome devaluation and action–outcome contingency degradation tests. We found that cocaine pre-exposure had no impact on rats’ ability to learn about multiple action–outcome relationships or use these associations when adapting to a change in reward value. Interestingly, rather than being impaired, cocaine-exposed rats displayed enhanced sensitivity to instrumental contingency degradation training. Thus, in a behavioral scenario that discourages habitual control, repeated cocaine exposure actually enhances certain features of flexible goal-directed behavior, which has important implications for our understanding of the neural and behavioral substrates of drug addiction.

## Materials and Methods

### Subjects and Apparatus

Adult male Long–Evans rats (*n* = 30) weighing ~375 g at the start of the experiment were used as subjects. Rats were pair-housed and had *ad libitum* access to water throughout the experiment. Rats had unrestricted access to food during the cocaine sensitization and withdrawal phases of the experiment but were maintained at ~85% of their free-feeding body weight during the following behavioral phases.

Behavioral procedures took place in Med Associates (St Albans, VT, USA) operant chambers located in sound- and light-attenuated cubicles. The chambers were equipped with four photobeams for monitoring locomotor activity across a horizontal plane ~2 cm above a stainless steel grid floor. Each chamber was also equipped with two retractable levers positioned to the right and left of a food magazine, which was mounted on the right end wall. Two pellet dispensers connected to the magazine and were used to deliver either plain (i.e., grain) or chocolate-flavored purified dustless precision pellets (45 mg, BioServ, Frenchtown, NJ, USA). The hind wall and the hinged front door were made out of transparent Plexiglas. A single houselight (3 W, 24 V) located on the left end wall illuminated the chamber.

During the cocaine sensitization phase of the experiment, we added visual, tactile, and olfactory cues to the bare chamber described above in order to create a distinctive context. Panels with vertical black-and-white stripes were positioned outside the transparent hind wall and front door; a white perforated Plexiglas sheet covered the grid floor; and 0.2 ml of pure almond extract (McCormick and Co. Inc., Baltimore, MD, USA) was poured directly into the stainless steel waste pan located under the grid floor.

All experimental procedures involving rats were approved by the UC Irvine Institutional Animal Care and Use Committee and were in accord with the National Research Council Guide for the Care and Use of Laboratory Animals.

### Drugs

Cocaine hydrochloride (Sigma-Aldrich, St. Louis, MO, USA) was dissolved in sterile saline (0.9%NaCl). Cocaine and saline (i.e., vehicle) solutions were injected i.p. at the volume of 1 ml/kg.

### Cocaine Exposure Protocol

To establish basal locomotor responding, all rats were first given a single injection of saline and were immediately placed in the operant chambers, where photobeam breaks were recorded for 60 min. Rats were then divided into three groups: two cocaine groups receiving cocaine injections at either 15 or 30 mg/kg, and one saline group (all *n*’s = 10) receiving saline injections. Rats were injected once daily for 15 consecutive days. Immediately after each injection, the rats were placed in the behavioral chambers (with modified context as described above) for 60 min during which locomotor activity was recorded. All rats remained undisturbed in their home cages for a further 29 days before being put on food restriction for subsequent behavioral testing.

### Instrumental Training

Starting on withdrawal day 32, rats received magazine training for 2 days. In each session, they received 20 grain and 20 chocolate food pellets randomly delivered on a random time (RT) 30 s schedule while the levers were retracted. Rats were then given 10 days of instrumental training on two distinct action–outcome contingencies (i.e., R1 → O1 and R2 → O2). Training with the right and left levers was carried out in two separate sessions each day. The specific lever-outcome arrangements were counterbalanced with drug treatment conditions, such that for half of the rats in each treatment group right lever pressing was paired with the delivery of the chocolate pellet while left lever pressing earned the grain pellet, whereas the other half received the opposite arrangement. During each session, only one lever was extended. The session was terminated after 30 min elapsed or 20 pellets were earned. The two daily sessions were separated by at least 2 h, and their order was alternated every day. For the first 2 days of the instrumental training phase, lever pressing was continuously reinforced (CRF). Instrumental training under a random ratio (RR) as opposed to a random interval schedule of reinforcement is known to discourage the emergence of habitual control over reward seeking (41). Because our study looked specifically at the effect of cocaine on goal-directed control, the schedule of reinforcement were gradually shifted to an RR-5 schedule for the next 2 days (i.e., lever presses resulted in a pellet delivery with *p* = 0.2), followed by an RR-10 schedule (*p* = 0.1) for an additional 2 days, and finally to an RR-20 (*p* = 0.05) for the last 2 days of the instrumental training.

### Devaluation Testing

In order to selectively diminish the value of one food outcome, relative to the other, all rats were allowed to become satiated on grain or chocolate pellets by providing them with 60 min of unrestricted access to that food (25 g/rat placed in a bowl, counterbalanced with the drug treatment conditions) in the home cage. Immediately following home cage pre-feeding (induction of specific satiety), rats underwent a devaluation test to assess their tendency to perform the two lever-press responses. Rats had continuous access to both levers throughout the test. Each test began with a 5-min extinction phase, during which lever pressing was recorded, but was not reinforced, which was done to assess response tendencies in the absence of explicit feedback. This was immediately followed by a 15-min reward phase, during which each response resulted in the delivery of its respective outcome according to CRF (for the first 5 pellets) and RR-20 (for the remainder of the test) schedules of reinforcement. On the following experimental day, rats underwent instrumental retraining sessions identical to the instrumental sessions described above, with the exception that the schedule of reinforcement shifted from CRF to RR-20 within the session (three pellets at CRF, two pellets at RR-5, one pellet at RR-10, and the remainder at RR-20). Retraining sessions lasted 30 min or were terminated after the delivery of 20 pellets. On the following day, all rats were given a second outcome devaluation test with the opposite outcome devalued. The order according to which each outcome was tested in a devalued state was counterbalanced between animals and treatment groups. Data presented are the average responses on devalued and non-devalued outcomes from the two testing days.

### Action–Outcome Contingency Degradation

#### Training

Following a day of instrumental retraining (same as during devaluation testing), rats underwent a contingency degradation protocol during which each lever-press action continued to produce its original pellet outcome on a modified RR-20 schedule commonly used in such studies (9, 16, 39, 42). Specifically, sessions were divided into a series of 1-s periods and the first press that was performed in each periods had a 1-in-20 chance of producing reward [p(O/A) = 0.05]. As before, the two actions were trained in separate daily sessions, though these sessions were now limited to 20 min and did not have a limit on the number of rewards that could be earned. Most importantly, however, during this phase of the experiment, one of the two pellets was additionally delivered in a non-contingent manner. Specifically, during each 1-s period without a lever-press response, either grain or chocolate pellets were delivered with the same probability that they would have been delivered following performance of the appropriate response [p(O/no A) = 0.05], thus degrading this action–outcome contingency. This outcome was delivered non-contingently during both daily contingency degradation training sessions, regardless of which lever was being trained. For degraded sessions, the non-contingent outcome was the same as that which was earned by a response on the available lever, whereas for non-degraded sessions, the non-contingent outcome was different from the earned outcome. Consequently, the non-contingent outcome could be expected with the same probability whenever the rat was placed in the behavioral chamber, regardless of whether they lever pressed or not. In contrast, the alternative outcome could only be obtained by performing the non-degraded action. Grain pellets were non-contingently delivered for half of the rats (counterbalanced with action–outcome contingency and drug treatment conditions), whereas the remaining rats received non-contingent chocolate pellets.

#### Testing

After 5 days of contingency degradation training, all rats underwent a 5-min choice extinction test, during which both levers were made available (Test 1). Lever presses were continuously recorded but did not produce any outcomes nor were any outcomes delivered non-contingently. Rats then received an additional 5 days of contingency training, followed by a second 5-min extinction test (Test 2).

### Data Analysis

Data were analyzed using mixed-design analysis of variance (ANOVA). Drug treatment was a between-subjects variable. Within-subjects variables included treatment day for the cocaine sensitization, training day for the instrumental training, outcome value for the devaluation tests, contingency and training day for the contingency degradation training, and contingency for the contingency degradation extinction test. When Mauchly’s test indicated that the assumption of sphericity had been violated, we used the Greenhouse–Geisser correction. To examine the source of interactions, Dunnett’s *post hoc* tests were used to assess group differences in the simple effects of Devaluation or Degradation (i.e., the difference in response rates across the two actions) and individual one-way ANOVAs were conducted to assess within-subjects effect. We also assessed group differences in choice of Devalued (or Degraded) actions during these tests, calculated as a percentage of total lever presses [Action 1/(Action 1 + Action 2) × 100]. Because these data had a binomial distribution, they underwent arcsine transformation before we analyzed them using a one-way ANOVA followed by Dunnett’s *post hoc* testing, when appropriate. We also conducted one-sample *t*-tests against the test value of 50% (i.e., no preference on either lever) for each group.

## Results

### Locomotor Sensitization

**Figure 1:**
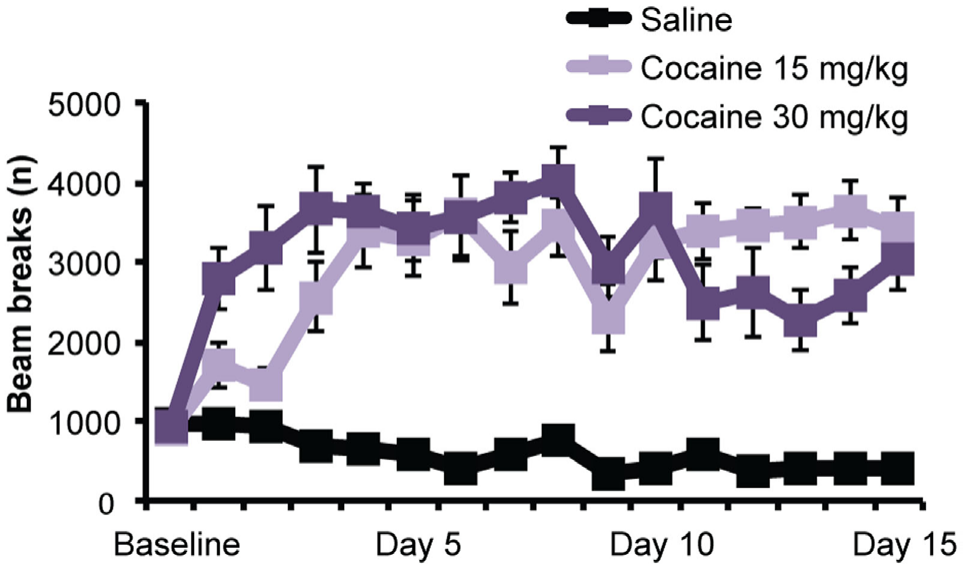
**Cocaine sensitization**. Chronic exposure to cocaine (15 and 30 mg/kg) significantly increased locomotor activity over days. Photobeam breaks mean (±SEM).

To assess baseline locomotor activity, all rats were given a single injection of saline before being placed in the behavioral chamber. No effect of Treatment group was detected [*F*(2,27) = 0.40; *p* = 0.68], indicating that basal activity did not differ between groups. However, as shown in Figure 1, subsequent cocaine treatment did significantly increase locomotor activity over days, relative to saline treatment. A mixed ANOVA (Day × Treatment) detected a significant main effect of Day [*F*(6.63,178.98) = 2.99; *p* < 0.01], a main effect of Treatment [*F*(2,27) = 42.79; *p* < 0.001], and a Day × Treatment interaction [*F*(13.26, 178.98) = 3.91; *p* < 0.001]. To further explore this interaction, we performed repeated-measures ANOVAs on the locomotor activity for each treatment group. Whereas this confirmed a significant increase in activity over days in cocaine-treated rats [*F*(4.27,38.41) = 4.17 and *F*(4.98,44.85) = 2.73; *p*’s < 0.05 for cocaine 15 and 30 mg, respectively], the analysis showed that saline-treated rats displayed a gradual decrease in activity [*F*(3.84,34.5) = 12.08; *p* < 0.001], indicating habituation to the context.

### Instrumental Training

**Figure 2:**
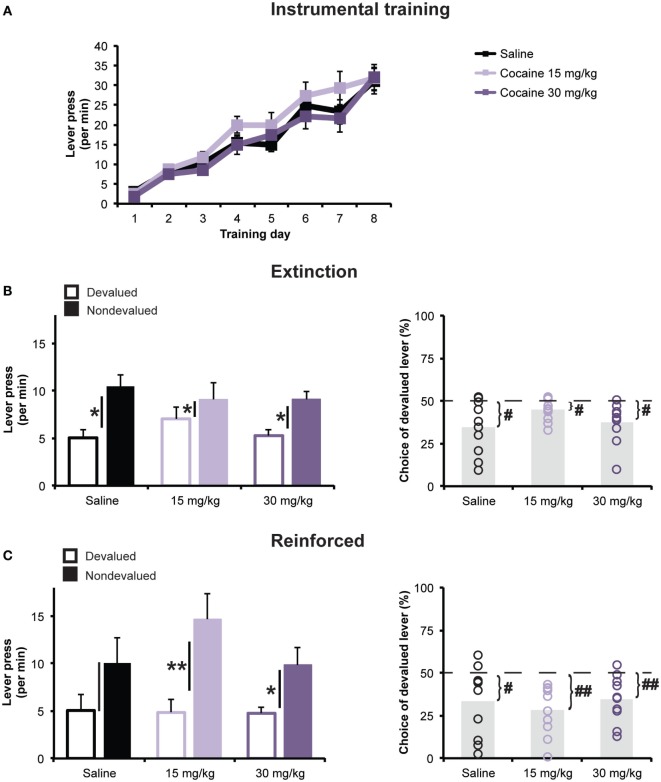
**Outcome devaluation**. **(A)** Daily mean (±SEM) rates of lever pressing (presses per minute) during the 8-day of instrumental training following repeated cocaine or saline exposure. **(B)** Responses during the 5-min extinction phase of the outcome devaluation test. *Left panel:* mean lever press rate (±SEM) on Devalued and Non-devalued levers. Black bars represent ±SEM of within-subject difference score (Non-devalued–Devalued). *p < 0.05 Devalued vs. Non-devalued. *Right panel:* mean (±SEM) percentage of all lever presses performed on the Devalued lever. ^#^*p* < 0.05 vs. 50%. **(C)** Responses during the 15-min reinforced phase of the outcome devaluation test on Devalued and Non-devalued levers. Black bars represent ±SEM of within-subject difference score (Non-devalued–Devalued). *p < 0.05 and **p < 0.01 Devalued vs. Non-devalued. *Left panel:* mean lever press rate (±SEM). *Right panel:* mean (±SEM) percentage of all lever presses performed on the Devalued lever. ^#^*p* < 0.05 and ^##^*p* < 0.01 vs. 50%.

Averages rate of responding on the two levers for the 8 days of instrumental training are presented in Figure 2A. Rats in all treatment groups rapidly acquired lever pressing and increased their response rates as the ratio schedule requirements were augmented. Statistical analysis revealed that the cocaine treatment had no effect on the acquisition of lever pressing during the training phase. A mixed ANOVA (Day × Treatment) revealed a significant main effect of Day [*F*(2.94,79.45) = 94.18; *p* < 0.001], but found no effect of Treatment [*F*(2,27) = 0.81; *p* = 0.45], or Day × Treatment interaction [*F*(5.88, 79.45) = 0.87; *p* = 0.52].

### Outcome Devaluation

Outcome devaluation testing was then conducted to assess the degree to which the rats could flexibly modify their choice between the two lever-press actions following a selective reduction in the incentive value of one of the two reward outcomes, accomplished using a sensory specific satiety procedure. Data presented in Figures 2B, C represent the average lever press rate during the two devaluation tests (see Materials and Methods for details).

#### Extinction Phase

During the first 5 min of each devaluation test, the two levers were present but did not result in outcome delivery. All three groups showed a reduction in their performance of the action whose outcome was currently devalued (Devalued action), relative to the action whose outcome was non-devalued (Non-devalued action), demonstrating that, regardless of drug treatment, all groups exhibited the capacity to use action–outcome learning to adapt their food-seeking behavior in a goal-directed manner. Supporting this interpretation, a mixed ANOVA (Devaluation × Treatment) revealed a significant main effect of Devaluation [*F*(1,27) = 26.33; *p* < 0.001], but found no main effect of Treatment [*F*(2,27) = 0.15; *p* = 0.86], or Devaluation × Treatment interaction [*F*(2,27) = 1.79; *p* = 0.19]. We went on to look at the effect of devaluation at the group level. Paired *t*-tests revealed a significant effect of the devaluation procedure on lever pressing for each treatment group (*t*’s < −2.5). Furthermore, when looking at the percentage of total presses directed toward the Devalued action (Figure 2B), a one-way ANOVA showed no significant differences between groups [*F*(2,27) = 1.41; *p* < 0.05]. For all groups, the Devalued action was chosen at a significantly lower rate than would be expected by chance (i.e., 50%; all *t*’s < −2.84), indicating a preference for the Non-devalued action.

#### Reinforced Phase

During the last 15 min of each devaluation test, both levers were reinforced with their respective outcomes according to an RR-20 schedule (Figure 2C). Here too, all groups exhibited a selective reduction in lever pressing for the devalued outcome, relative to the alternate action. A mixed ANOVA detected a significant main effect of Devaluation [*F*(1,27) = 22.38; *p* < 0.001], but found no main effect of Treatment [*F*(2,27) = 0.73; *p* = 0.49], or Devaluation × Treatment interaction [*F*(2,27) = 1.2; *p* = 0.32]. As during the extinction test, choice of the Devalued action (% of total press) did not significantly differ among groups [*F*(2,27) = 0.44; *p* > 0.05], and all groups displayed significantly preference for the Non-devalued action (all *t*’s < −2.49).

### Contingency Degradation

#### Training

**Figure 3:**
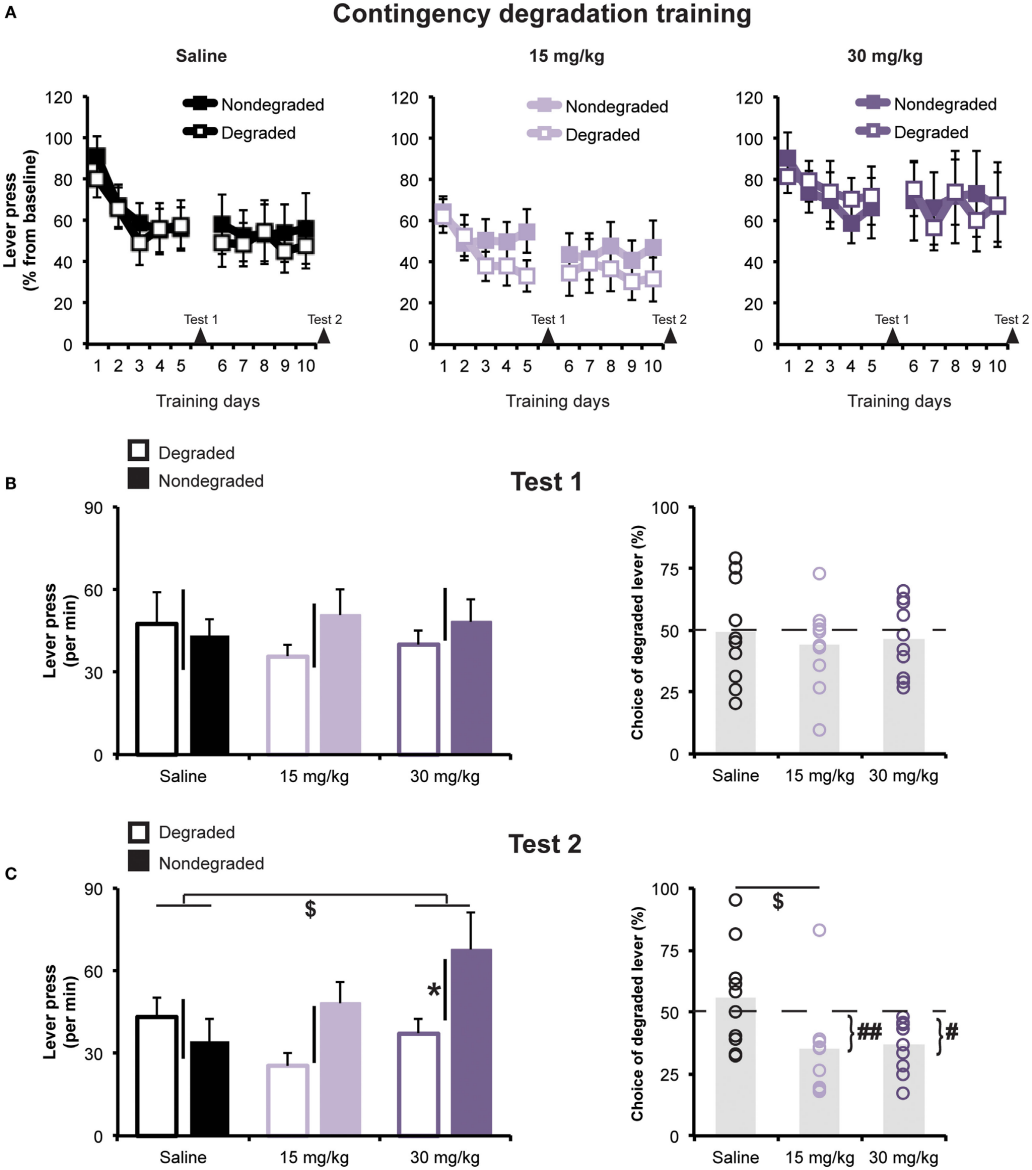
**Contingency degradation (A) Mean (±SEM) rates of lever pressing during contingency degradation training**. Lever pressing rates are expressed as % of baseline. Arrows indicate test days **(B)** responses during Extinction Test 1. *Left panel:* mean press rate (±SEM) on lever for which the contingency was Degraded or Non-degraded. Black bars represent ±SEM of within-subject difference score (Non-degraded–Degraded). *Right panel:* mean (±SEM) percentage of all lever presses performed on the Degraded lever. **(C)** Responses during Extinction Test 2. *Left panel:* mean press rate (±SEM) on lever for which the contingency was Degraded or Non-degraded. *p < 0.05 Degraded vs. Non-degraded. ^$^*p* < 0.05 vs. saline-treated group. Black bars represent ±SEM of within-subject difference score (Non-degraded–Degraded). *Right panel:* mean (±SEM) percentage of all lever presses that were performed on the Degraded lever. ^$^*p* < 0.05 vs. saline-treated group. ^#^*p* < 0.05 and ^##^*p* < 0.01 vs. 50%.

Next, we investigated the effects of cocaine treatment on rats’ capacity to adjust their instrumental food-seeking behavior to accommodate a selective reduction in action–outcome contingency. Figure 3A shows the rats’ average response rates during contingency degradation training sessions, plotted separately for each treatment group, for the action whose outcome was non-contingently presented (Degraded action) and for the alternate action (Non-degraded action), whose outcome was only delivered in a response-contingent manner. Data are expressed as percentage of performance from the instrumental training baseline (i.e., last day of instrumental retraining), whose values are presented in Table 1. A mixed ANOVA conducted on these data found no effect of Treatment [*F*(2,27) = 0.42; *p* = 0.66], or Degradation (to-be Degraded vs. to-be Non-degraded; *F*(1,27) = 0.0; *p* = 0.99), and found no evidence of a pre-existing Treatment × Degradation interaction [*F*(2,27) = 1.37; *p* = 0.27].

**Table 1:** **Instrumental training baseline**.

Treatment	Lever presses		*p*-Value
	To-be degraded	To-be non-degraded	
Saline	34.79 (±5.79)	38.69 (±7.40)	0.17
Cocaine 15 mg/kg	46.51 (±6.71)	43.33 (±6.67)	0.30
Cocaine 30 mg/kg	39.92 (±6.93)	39.14 (±6.13)	0.83

*Summary of the mean (±SEM) rate of lever pressing (presses per minute) during the last day of instrumental training (RR-20 schedule of reinforcement) before the start of contingency degradation training*.

Figure 3A shows the results of contingency degradation training. As is frequently the case in such experiments (39, 42, 43), we did not observe any response-specific effect of the non-contingent reward delivery during contingency degradation training sessions, though we did observe a general decline in response rates over days, an effect that was similar for all groups. A mixed ANOVA (Day × Degradation × Treatment) detected a significant effect of Day [*F*(2.7,73.04) = 3.47; *p* < 0.05], but found no effect of Degradation or Treatment [*F*(1,27) = 1.71; *p* = 0.2, and *F*(2,27) = 0.2; *p* = 0.82, respectively]. Nor were there any significant interactions (greatest *F* value = 1.97; *p* > 0.15).

#### Testing

Non-contingent rewards are known to have acute action-biasing effects on instrumental performance (15, 44–47) that can oppose and potentially obscure the expression of contingency degradation learning (48, 49). Therefore, our primary test of sensitivity to contingency degradation involved assessing rats’ choice between the two lever-press actions in a choice extinction test. An initial test administered between contingency training sessions 5 and 6 (Test 1; see Figure 3B) found no Degradation effect [*F*(1,27) = 0.82; *p* = 0.37], Treatment effect [*F*(2,27) = 0.05; *p* = 0.95], or Degradation × Treatment interaction [*F*(2.27) = 0.65; *p* = 0.53]. Choice of the Degraded lever (percentage of total lever presses; see Figure 3B) did not differ between groups [one-way ANOVA, *F*(2,27) = 0.22; *p* > 0.05], and no groups exhibited a preference that significantly differed from chance (i.e., 50%, all *t*’s > −0.2). However, when rats were re-tested following contingency degradation session 10 (Test 2; Figure 3C), we found that cocaine-treated groups had learned to selectively reduce their performance of the Degraded action. A mixed ANOVA detected a significant main effect of Degradation [*F*(1,27) = 5.59; *p* = 0.03], but found no effect of Treatment [*F*(2,27) = 1.79; *p* = 0.19]. More importantly, however, there was a significant Degradation × Treatment interaction [*F*(2,27) = 3.68; *p* = 0.04], indicating that the groups differed in their choice between the two actions. Interestingly, the Degradation effect was significant for the group given repeated exposure to the high dose of cocaine (*p* = 0.02), but was not significant, according to paired *t*-tests, for saline-treated rats (*p* = 0.44), or for rats treated with the low dose of cocaine (*p* = 0.06). Moreover, *post hoc* analysis on the responses difference score showed that the group treated with cocaine 30 mg/kg significantly differed from the saline-treated group (Dunnett’s test; *p* < 0.05). However, analysis of choice measure found evidence of contingency learning for the group given exposure to the low dose of cocaine. An ANOVA revealed a significant effect of Group [*F*(2,27) = 4.19; *p* < 0.05], and *post hoc* Dunnett’s test found that the group treated with 15 mg/kg, but not 30 mg/kg, cocaine significantly differed from the saline-treated group (*p*’s = 0.02 and 0.07, respectively). Only the two cocaine-treated groups chose the Degraded action significantly below levels that would be expected by chance (50%; *t*’s < −3.15, while for the saline-treated group, *t* = 0.64).

## Discussion

The current study examined the effects of repeated cocaine exposure on adaptive goal-directed behavior under conditions that discourage habitual control. Rats pre-treated with cocaine exhibited normal sensitivity to outcome devaluation, demonstrating that they had encoded the two action–outcome relationships and were unimpaired in using this information when adapting to an acute, outcome-specific reduction in the value of a behavioral goal. Interestingly, cocaine-treated rats displayed augmented – rather than impaired – sensitivity to action–outcome contingency degradation.

These findings would seem to be at odds with a vast body of data indicating that chronic exposure to cocaine or other abused drugs can bias adaptive behavioral control in favor of habits (25–29, 50). Nelson and Killcross (25), for instance, were the first to show that rats given repeated experimenter-administered amphetamine injections prior to learning to lever press for food reward developed devaluation-insensitive (habitual) performance under limited-training conditions that support devaluation sensitive performance in drug-naive rats. Repeated cocaine pre-exposure is known to have a similar habit-promoting effect (27, 29, 50, 51). Such findings are consistent with the view that pathological behaviors observed in addiction, and animal models of cocaine seeking, reflect an excessive reliance on automatic, inflexible response selection (3–5, 7).

An important question raised by such findings is whether this overreliance on habits is caused by an enhancement in habit-related processes or if it is simply a compensatory response to dysfunction in goal-directed processes. Some insight into this issue was provided early on by Nelson and Killcross (25), who found that instrumental performance remained goal-directed (devaluation sensitive) when rats were exposed to amphetamine after initial training but before testing. This result suggests that the drug-induced bias toward habitual performance that Nelson and Killcross observed when rats were exposed to amphetamine prior to training was caused by an enhancement of habit *formation* and not a disruption of goal-directed control. However, it is worth noting that LeBlanc et al. (27) found that rats previously exposed to cocaine displayed insensitivity to food outcome devaluation even when they were given response-contingent reinforcement at test, which is remarkable because normal (drug naive) rats are known to rapidly re-exert goal-directed control over their behavior under such conditions (4). Consequently, this finding could reflect a deficit in goal-directed control or at least the acquisition of habits that resist transition back to goal-directed control.

It is important to emphasize that most studies on this subject, whether investigating the effect of repeated amphetamine [e.g., Ref. (25)] or cocaine treatment [e.g., Ref. (27, 29)], have employed relatively simple instrumental tasks that provide subjects with only one reward option. Although this approach is useful for studying habit formation, it is not optimal for assessing the integrity of goal-directed learning and decision-making processes. As just discussed, when this approach is used, performance that is insensitive to outcome devaluation may either reflect an overreliance on habitual control, or a failure to properly encode or use the detailed action–outcome representations needed to respond in a goal-directed manner. Another problem with this approach is that it is more susceptible to concerns about the role of incidental Pavlovian learning in expression of task performance. There is evidence that Pavlovian context-reward learning can facilitate instrumental reward seeking (52), and that the strength of its influence is sensitive to changes in physiological need state (53, 54). Such findings support the long-standing view that Pavlovian learning processes contribute to the motivational control of instrumental behavior (55). Consistent with this, it was recently shown (56) that when rats are given limited training on a simple (one reward) lever-press task, it is possible to eliminate the sensitivity of instrumental performance to outcome devaluation by extinguishing the training context prior to testing. Such findings suggest that, for instrumental tasks involving only one reward option, outcome devaluation performance may be largely mediated by stimulus–outcome rather than action–outcome learning.

These concerns can be avoided by using a more complex instrumental decision-making task, such as the one used in the current study, in which animals are allowed to choose between two distinct reward-motivated actions. Although poorly understood, it is known that decision-making scenarios such as this discourage the acquisition of habitual control (30, 33, 34). Interestingly, it has been shown that rats can develop response-specific habits when given extensive training with one of two distinct action–outcome contingencies [e.g., Ref. (57)]. However, in such studies, each action is trained and tested in a unique context. In contrast, rats given extensive training with two action–outcome contingencies in a common context fail to develop habitual performance (30, 33). This has been observed even when rats are given a choice between responses during training and test sessions (34), which suggests that contextual changes across phases of the experiment (i.e., shifting from training sessions with only one response to test sessions in which two responses are available) are not primarily responsible for disrupting habitual performance during choice tests. Although more research is needed to characterize the psychological and neurobiological mechanisms that arbitrate between habitual and goal-directed action selection strategies, such findings suggest that having a choice between distinct response options at test is an important factor that biases behavioral control in favor of the latter. Assessing goal-directed control in rats trained (and tested) on two action–outcome contingencies in a common context also has another practical benefit in terms of data interpretation. Because the test context is associated with both the devalued and non-devalued reward, it alone (i.e., as a Pavlovian cue) is unlikely to provide the kind of reward-specific information needed to support differential action selection based on expected reward value.

For these reasons, two-option choice tasks provide a more direct approach for assaying goal-directed learning and action selection. Therefore, the current findings provide strong evidence that goal-directed processes are largely spared following repeated exposure to cocaine, at least for the drug exposure regimens tested here. As animals here were passively exposed to cocaine, our study did not address whether chronic cocaine self-administration also spares goal-directed decision-making nor does the current study speak to whether rats come to rely on a habitual or goal-directed strategy when seeking or taking cocaine. However, our findings may shed light on a recent study investigating changes in behavioral control over cocaine self-administration. It is known that rats given extensive opportunity to self-administer cocaine tend to develop a compulsive pattern of intake characterized by an insensitivity to response-contingent punishment (1, 2, 58). More recently, however, it was shown that providing rats with concurrent access to an alternative response option (sugar self-administration) attenuates the development of compulsive cocaine seeking under these exposure conditions (59). This fits nicely with the current results and further suggests that two-option scenarios such as the one used here promote goal-directed decision-making over habitual control. However, further research will be needed to more directly test this hypothesis.

Because our aim was to investigate the long-term behavioral effects of this treatment, we used a relatively lengthy (15-day) cocaine exposure regimen that included both intermediate (15 mg/kg) and high (30 mg/kg) drug doses, followed by a relatively lengthy (32-day) interval between drug exposure and the initiation of behavioral training for food. This is notable because previous findings of drug-induced facilitation of habit formation have typically used shorter drug exposure (6–10 days) and exposure-to-training intervals (7–14 days). Such procedural differences, however, are unlikely to explain our findings given that cocaine exposure regimens similar to those used here are known to be effective in causing persistent alterations in reward-motivated behavior (51, 60). For instance, Schoenbaum and Setlow (51) found that cocaine-treated rats (14 injections; 30 mg/kg) given a 21-day withdrawal period before training on a simple food-motivated Pavlovian approach task developed rigid conditioned approach behavior that was insensitive to reward devaluation. Furthermore, using a two-option task such as the one used here, LeBlanc (37) found normal sensitivity to outcome devaluation in rats pre-treated with a shorter cocaine exposure regimen known to facilitate habit formation (27).

The study by LeBlanc (37) is one of very few that has assessed the effects of repeated drug exposure on adaptive goal-directed behavior using a two-option choice task that discourages habit formation. Another such study (38) found that rats given repeated amphetamine injections prior to training also showed normal sensitivity to reward devaluation during a two-option choice test. Together with the current results, such findings suggest that although chronic experience with psychostimulant drugs can profoundly alter adaptive behavior, this is not related to generalized hypofunction in neural systems underlying goal-directed behavior. That said, recent studies have shown that alcohol- and methamphetamine-associated contextual cues are effective in disrupting goal-directed choice between different reward options (61, 62), suggesting that Pavlovian stimulus-drug learning may contribute to drug-induced behavioral dysregulation. Importantly, this possibility was not investigated in the current study, as rats were exposed to cocaine in the presence of contextual cues that were clearly discriminable from those present during instrumental training and testing and were repeatedly handled and exposed to the main behavioral apparatus (without further cocaine exposure) prior to testing, which likely extinguished any unintended drug-related learning that happened to occur.

Our finding that repeated cocaine exposure heightened rats’ sensitivity to action–outcome contingency degradation demonstrates that the cocaine regimen used here was, in fact, effective in altering goal-directed processes, albeit in a manner that is at odds with the view that cocaine exposure disrupts goal-directed control. However, this finding was not entirely unanticipated. Though few in number, studies assessing the impact of chronic drug exposure on this aspect of learning have observed similar effects (39, 40). Most relevant to the current study, Phillips and Vugler (39) used a two-option task, similar to the one used here, to investigate the effects of a sensitizing regimen of amphetamine injections on contingency degradation learning. They found that amphetamine-treated rats displayed enhanced sensitivity to contingency degradation, in that they selectively suppressed their performance of an action that was no longer needed to produce its outcome, an effect that emerged well before it did in saline-treated rats (39). It should be noted that, in this study, amphetamine-treated rats did not significantly differ from saline-treated rats during a final (non-reinforced) choice test. However, because this test was conducted after both groups displayed evidence of contingency sensitivity during training sessions, it was not likely to reveal group differences in the *rate* of contingency degradation learning. This was not an issue in the current study since we conducted choice extinction tests before saline-treated rats showed evidence of contingency degradation learning, an effect that can require many sessions of training to emerge in some studies (39, 43), and which may have been particularly slow to develop for the task used here due to our use of highly similar reward options.

The differential effects of cocaine exposure on devaluation and contingency testing suggest that this drug treatment does not augment goal-directed learning or control in a general way. Instead, it is possible that this finding reflects a fundamental alteration in the way animals adapt to changes in action-reward contingencies. For instance, it has been shown that cocaine-treated rats’ exhibit heightened sensitivity to differences in reward delay and magnitude when deciding between reward options (60). Another possibility is that cocaine exposure alters processes specific to contingency degradation learning, including the ability to track information about non-contingent reward deliveries and integrate this with information about response-contingent reward probabilities. Because non-contingent rewards occur in the absence of other, more predictive cues, it is believed that the likelihood of their occurrence is tracked through context conditioning (63, 64). This view assumes that the probability that an instrumental action will be performed depends on its ability to serve as a reliable predictor of reward, relative to other potential predictors, including contextual cues. Given this competition, the rate at which an action is performed should be inversely related to the degree to which the test context predicts the delivery of the reward earned by that action. From this perspective, the key to understand cocaine’s impact on contingency learning may be related to its well-established facilitative influence on Pavlovian (stimulus-reward) learning (27, 65–70), since this should allow the context to better compete with instrumental actions for cocaine-treated rats.

Drug-induced enhancement in stimulus-reward learning has been linked to hyper-responsivity in ascending dopamine systems (70, 71). This is interesting given the finding that dopamine-depleting lesions of the dorsomedial striatum disrupt rats’ sensitivity to action–outcome contingency degradation but spares their ability to select between actions during outcome devaluation testing (72), even though this structure is known to be a key mediator of both of these features of goal-directed behavior (41). There is, in fact, quite strong evidence that dopamine transmission is not critical for the instrumental incentive learning process responsible for encoding changes in value of rewards or in using such information to control instrumental goal-directed behavior (31, 73), which may explain our finding that these processes were relatively unaffected by repeated cocaine exposure. Interestingly, Corbit et al. (29) recently found evidence that a habit-facilitating cocaine exposure regimen augmented presynaptic glutamate signaling in the DMS. While it was suggested that this phenomenon could reflect a state of DMS dysfunction, leading to impaired goal-directed control, we suggest that it may also contribute to the augmented contingency degradation effect reported here.

It remains unclear if drug-induced augmentation of instrumental contingency degradation learning is a harmless side effect of drug intake or if it contributes in some way to the addiction process. For example, it has been suggested that some individuals may use psychostimulants in order to cope with poor cognitive performance associated with pathologies, such as attention deficit and hyperactivity disorder (ADHD). Interestingly, it was recently shown that treatment with the psychostimulant methylphenidate could restore certain features of goal-directed control in a rat model of ADHD (74). However, the immediate beneficial effects of such drugs may lead to drug abuse and addiction. For instance, it is believed that the use of psychostimulants for self-medication purposes could be an important contributor to the high comorbidity rate of ADHD and substance use disorder (75). Alternatively, it is possible that the augmentation of goal-directed contingency learning following chronic cocaine exposure may actually have disruptive effects on behavioral control that were not observed in the current study. For instance, it has been suggested that in some circumstances, chronic drug intake may disrupt the development of adaptive habits for routine tasks (40), which could overburden the goal-directed system and impair decision-making when cognitive resources become taxed. The hypothesis that drug exposure disrupts behavioral flexibility by misallocating cognitive resources should be explored further, as it could have important implications for addiction theory and research.

## Author Contributions

BH contributed to experimental design and execution, performed statistical analysis and generated figures, and participated in the writing of the manuscript. AL contributed to experiment design and execution and assisted with data analysis. SO contributed to experimental design, data analysis, and manuscript preparation. All authors read, provided feedback, and approved the final version of the paper.

## Conflict of Interest Statement

The authors declare that this research was conducted in the absence of any commercial, financial, or other relationships that could be construed as a potential conflict of interest.
